# Diagnostic dilemma of lobular carcinoma: a mini-review of imaging modalities and the role of artificial intelligence and radiomics

**DOI:** 10.3389/fonc.2025.1515037

**Published:** 2025-03-27

**Authors:** Sima Jabbari, Mariam Shadan, Yousef Eltayeb, Omer El Faroug Salim, Nimrah Afaq, Rushda Haider, Sana Sohail Shaikh

**Affiliations:** Dubai Medical College for Girls (DMCG), Dubai Medical University, Dubai, United Arab Emirates

**Keywords:** breast imaging, AI, radiomics, breast specific gamma imaging (BSGI), digital breast tomosynthesis (DBT), ultrasound, magnetic resonance imaging (MRI), mammography (MeSH)

## Abstract

Lobular carcinoma (LC) presents unique diagnostic challenges due to its subtle imaging characteristics and asymptomatic presentation, often leading to delays in diagnosis and treatment. This mini-review critically examines both traditional and advanced imaging modalities used to detect and manage LC, including mammography, ultrasound, digital breast tomosynthesis (DBT), contrast-enhanced mammography (CEM), breast magnetic resonance imaging (MRI), and breast-specific gamma imaging (BSGI). Traditional modalities like mammography and ultrasound, while widely used, have limitations, particularly in detecting LC in patients with dense breast tissue. Advanced techniques, such as MRI and BSGI, offer improved sensitivity and specificity but are limited by cost and accessibility. Emerging technologies such as artificial intelligence (AI) and radiomics are reshaping the diagnostic landscape for LC. AI has shown promise in enhancing diagnostic accuracy, predicting treatment outcomes, and improving risk stratification by analyzing large datasets from multiple sources, including imaging, genomic, and clinical data. Radiomics, which extracts quantitative features from medical images, further complements AI by providing detailed insights into tumor characteristics, treatment responses, and molecular subtypes of breast cancer, including LC. Together, AI and radiomics have the potential to revolutionize the detection, characterization, and monitoring of LC, particularly by enhancing the accuracy of traditional imaging methods and supporting personalized treatment strategies. This review also provides actionable recommendations for clinicians, radiologists, and researchers on the integration of advanced imaging techniques and AI into clinical workflows. With continued advancements, AI and radiomics are poised to improve the early detection and management of LC, ultimately contributing to better patient outcomes.

## 1 Introduction

Invasive lobular carcinoma (ILC) is the second most common type of breast cancer, accounting for approximately 10%–15% of all breast cancer cases (1). Unlike its counterpart, invasive ductal carcinoma (IDC), ILC is often more challenging to detect and diagnose due to its unique biological characteristics and growth patterns. ILC tends to infiltrate breast tissue in a diffuse and linear fashion, lacking the distinct masses or lumps that are typically associated with IDC. This subtle and diffuse infiltration often results in delayed diagnosis, making it critical to explore the full spectrum of diagnostic tools available (2).

Over the years, various imaging and diagnostic techniques have been employed to improve the detection and characterization of lobular carcinoma. Traditional methods such as mammography and ultrasound remain first-line approaches; however, the limitations of these modalities, particularly in cases of ILC, necessitate the inclusion of more advanced and specialized diagnostic tools. Magnetic resonance imaging (MRI), digital breast tomosynthesis (DBT), and molecular imaging techniques have emerged as valuable adjuncts in the evaluation of ILC (3). Additionally, biopsy methods—guided by both imaging and histopathology—remain essential for definitive diagnosis (1, 2).

This paper aims to provide an overview of the current diagnostic modalities available for the detection and diagnosis of invasive lobular carcinoma. By exploring both conventional and emerging technologies, we hope to elucidate the strengths and limitations of each modality and propose an integrative approach to improve the early detection and management of ILC.

## 2 Traditional imaging techniques

### 2.1 Mammography

Mammography is the most widely used screening tool (4) for breast cancer but has notable limitations in detecting LC due to its indistinct radiographic features, especially in dense breast tissue (5). Although mammography’s overall sensitivity is approximately 85%, it drops to 68% in women with dense breasts (6) leading to higher false negatives (7). Also, in contrast to ductal carcinoma, lobular carcinoma lacks the calcification that allows the lesion to escape detection, deserving to be called stealth phenomena (8). Tumor size is often underestimated in LC, with spiculated masses being the most common finding, while well-circumscribed masses are rare, occurring in less than 1% of cases (4).

### 2.2 Ultrasound

Ultrasound is often used as a supplementary tool to mammography, particularly in cases of dense breast tissue or palpable abnormalities (9, 10). While ultrasound is independent of breast density and radiation-free, its operator-dependent nature leads to variability in interpretation (11–13). It is also valuable for guiding biopsies and evaluating axillary lymph nodes (14, 15), but its effectiveness is limited by the skill of the operator and in multifocal lesions (16).

## 3 Advanced imaging techniques

### 3.1 Breast MRI

Breast MRI offers exceptional soft tissue contrast and is highly sensitive in detecting lesions, particularly in patients with dense breast tissue. MRI is especially useful in identifying single spiculated masses that correlate well with tumor pathology (4). However, its high cost, increased false-positive rate, and potential for overdiagnosis limit its widespread use (17). A study by Lee et al. (18) found that MRI was significantly more sensitive than ultrasound in detecting residual lobular carcinoma *in situ* (LCIS) after surgical excision, with a sensitivity of 83.3% compared to 58.3%. However, one significant concern is its relatively low specificity, which can lead to a higher rate of false positives. This issue is particularly problematic as it may result in unnecessary biopsies and increased patient anxiety (19). In a study, only 24.8% of MRI findings that were positive for cancer were confirmed upon biopsy, indicating a substantial rate of false-positive results (19). This can complicate the clinical decision-making process and lead to overtreatment.

### 3.2 Digital breast tomosynthesis

DBT is an advanced form of mammography that provides three-dimensional imaging, enhancing detection rates for subtle lesions such as LC (20). While DBT improves lesion visualization and offers greater sensitivity than standard mammography (20, 21), studies indicate that it is more effective in detecting invasive cancers like invasive lobular carcinoma (22). Despite these advantages, DBT is expensive and requires specialized equipment (23), additional radiation exposure (24), and breast compression.

### 3.3 Contrast-enhanced mammography

Contrast-enhanced mammography (CEM) combines full-field digital mammography with a dual-energy technique, using iodinated contrast to improve visualization of breast tissue (25). CEM has demonstrated higher diagnostic accuracy than traditional mammography, with faster interpretation times and greater accessibility compared to MRI (4, 26, 27). While it has been found to be non-inferior to MRI in some studies (28), MRI remains the most sensitive modality for detecting occult cancers, particularly in high-risk populations (29).

### 3.4 Breast-specific gamma imaging

Breast-specific gamma imaging (BSGI), also known as molecular breast imaging (MBI), uses technetium-99m sestamibi and a high-resolution gamma camera to detect breast malignancies (6). BSGI has shown a high sensitivity of 93%, detecting lesions that mammography might miss (30, 31), and has demonstrated higher specificity (81.44%) than other imaging methods (5). However, BSGI is less effective than MRI in identifying cancers in the axilla or chest wall (32), limiting its role as a supplemental tool rather than a primary screening modality.

## 4 Comparative insights: Traditional vs. advanced modalities

A direct comparison of the diagnostic capabilities of traditional and advanced imaging techniques shows that advanced modalities offer clear advantages. MRI provides the highest sensitivity among all modalities (33), especially for dense breast tissue, but its high cost and overdiagnosis potential remain concerns (17). DBT, though an improvement over mammography, is not specifically optimized for LC detection but has shown promise in detecting invasive lesions (22). CEM and BSGI both improve detection rates over traditional mammography, but BSGI stands out for its ability to detect lesions that are otherwise difficult to identify, making it an excellent tool for supplementary diagnostics. These findings are summarized in **Table 1**.

**Table 1:** Comparison of imaging modalities for the diagnosis of lobular carcinoma.

Modality	General advantages	LC-specific advantages	General disadvantages	LC-specific disadvantages
Mammography	Widely used, low cost (4)	Identifies architectural distortion and asymmetry associated with ILC (30)	False-negative rates are higher for ILC (7); sensitivity drops in dense breast tissue (5, 6)	May not detect ILC due to lack of desmoplastic response and subtle growth patterns (8, 30)
Ultrasound	Non-invasive, radiation-free, real-time imaging, useful for guiding biopsies	More sensitive than mammography in detecting ILC in dense breast tissue (9, 10)	Operator-dependent, variability in accuracy, particularly for subtle ILC (11–13)	May not visualize the full extent of ILC, especially multifocality (16)
Breast MRI	Exceptional soft tissue contrast and is highly sensitive in detecting lesions, particularly in patients with dense breast tissue (18)	Useful in identifying single spiculated masses (4)	High cost; increased false-positive rate (17, 19)	Potential for overdiagnosis (17, 18)
Digital breast tomosynthesis (DBT)	Provides 3D imaging, reduces superimposition of tissues, improves detection of small lesions (20)	Particularly helpful for detecting the subtle and infiltrative growth patterns of ILC (20, 21)	Expensive; requires specialized equipment (23); increased radiation (24)	Less effective for small, non-calcified cancers (21); may miss subtle features of ILC (34)
Contrast enhanced mammography (CEM)	Enhances sensitivity, particularly in dense breast tissue; faster than MRI; more accessible (25)	Enhanced detection of subtle ILC features, improves differentiation of malignant lesions (26, 27)	Requires iodinated contrast (potential for allergic reactions), higher cost, adds radiation exposure (35)	May not completely eliminate mammography’s false-negative issues for ILC (27)
Breast-specific gamma imaging (BSGI)	High sensitivity for detecting breast cancer; non-compressive imaging (5)	Can detect ILC, particularly in dense tissue; visualizes subtle changes in breast tissue (30, 31)	Potential for false positives, leading to unnecessary biopsies (32)	Less effective for chest wall or axillary cancers (32)
AI and radiomics	Quantitative analysis, non-invasive, integrates with AI for personalized treatment strategies	Helps in identifying subtle ILC features, provides insights into tumor behavior (36)	Complex data analysis, requires specialized computational tools, not widely adopted (37)	Requires rigorous validation for clinical use (38); may miss clinical factors important for diagnosis (39)

## 5 Future directions in LC diagnosis: AI and radiomics applications

The unique biological behavior and clinical characteristics of ILC present challenges in treatment planning and prognosis. However, AI and radiomics have shown promise in enhancing the diagnosis and risk stratification of ILC (40). While both radiomics and AI use imaging data to extract valuable insights, their approaches and applications differ. Radiomics involves the extraction of quantitative features from medical images, such as texture, shape, and intensity. These features provide detailed information about tumor characteristics that are not always visible to the human eye. Radiomics can enhance diagnostic accuracy, predict treatment responses, and help characterize tumors (36, 41). In contrast, AI integrates data from multiple sources—imaging, genomic, and clinical data—to perform predictive modeling and decision-making. AI uses these integrated datasets for tasks such as automated lesion detection, risk stratification, and prognosis, often incorporating radiomic features in its analysis. While radiomics focuses specifically on image-based features, AI combines multiple data types for broader clinical applications, including personalized treatment planning and long-term patient monitoring (42, 43).

Therefore, while radiomics provides detailed imaging data focused on tumor characteristics, AI goes further by combining imaging data with genomic and clinical information to make predictions and assist in clinical decision-making. AI can incorporate radiomic features into its analysis, but radiomics itself is focused on quantifying image-related tumor characteristics to inform diagnosis and prognosis.

Most AI-powered systems show promising results in improving diagnostic accuracy by analyzing large datasets from these imaging techniques, identifying subtle features in lobular carcinoma that may be missed by traditional methods (44, 45). A 2020 study by McKinney et al. evaluated an AI system for breast cancer screening across multiple international datasets. Their study revealed that the AI system outperformed human radiologists in identifying breast cancer, reducing false positives and false negatives, which is especially important for subtle and diffuse cancers like ILC (44). Similarly, Frazer et al. (45) evaluated deep learning-based AI techniques for breast cancer detection in mammograms and found that AI demonstrated high accuracy in breast cancer detection, with the potential to serve as a supplementary tool to radiologists in screening programs (45).

In addition to enhancing diagnostic accuracy, AI’s potential for improving diagnostic workflows in the context of LC includes aiding in risk stratification. For instance, AI models trained on imaging data from mammography, MRI, and ultrasound can analyze subtle features in lobular carcinoma, such as multifocality or bilaterality, which are crucial for accurate diagnosis and treatment planning (46, 47). By integrating AI into the diagnostic process, clinicians can make more informed decisions on the likelihood of disease recurrence and metastasis, thus refining risk stratification specifically for LC patients.

Moreover, AI enhances risk stratification by identifying patients at higher risk of recurrence or metastasis. Research suggests that ILC has a propensity for bilaterality and multifocality (43, 46). AI models can analyze imaging data to detect these features and help in more accurate risk assessment. For example, AI models trained on mammographic and MRI images can identify characteristics associated with a higher risk of contralateral breast cancer (47, 48).

Furthermore, AI can integrate clinical risk factors with molecular data to create comprehensive risk assessment tools. By incorporating a broader range of data, such as genetic mutations (e.g., CDH1 mutations) and hormonal factors, AI can provide more nuanced risk profiles (47, 48). This leads to more accurate predictions regarding disease progression and treatment outcomes.

AI’s ability to analyze histopathological data can also provide insights into tumor behavior and prognosis. For example, certain histological features, such as E-cadherin expression, are known to influence the aggressiveness of ILC (49). AI algorithms trained on histopathological images can identify these features and correlate them with clinical outcomes, enhancing prognostic accuracy (50).

In addition to AI, radiomics complements existing imaging by extracting quantitative features from medical images, offering deeper insights into tumor biology that traditional imaging may miss. It enhances diagnostic accuracy, improves risk stratification, and informs treatment decisions. For example, MRI-derived radiomic features can predict responses to neoadjuvant chemotherapy in lobular carcinoma patients (51), helping tailor personalized treatment plans.

Radiomics also plays a role in identifying breast cancer subtypes, aiding in the selection of therapeutic strategies (52), which is particularly useful for managing the subtle imaging characteristics of lobular carcinoma. Combined with AI, radiomics further enhances detection and characterization by analyzing vast datasets, offering improved sensitivity in dense breast tissue (53).

Moreover, radiomics helps monitor treatment responses and predict recurrence by tracking changes in tumor features, which is vital for managing lobular carcinoma’s varied growth patterns and therapy responses (54). When integrated with traditional imaging modalities like mammography and ultrasound, radiomics improves diagnostic workflows, enhancing the accuracy of interpretations and aiding in the distinction between benign and malignant lesions (55), leading to earlier detection and better outcomes.

## 6 Recommendations for clinicians and radiologists

1. Adopt multimodal imaging: Prioritize combining modalities such as MRI, DBT, and BSGI, particularly for high-risk or dense-breast patients, and incorporate this approach into clinical guidelines.
2. Leverage AI for diagnostic support: Use AI tools to enhance lesion detection, reduce false positives, and improve risk stratification. Ensure radiologists receive training to effectively use AI as a supplement to, not a replacement for, clinical expertise.
3. Collaborate on data integration: Work with multidisciplinary teams to establish systems that integrate AI insights across imaging modalities, supported by standardized data-sharing protocols.

## 7 Recommendations for researchers

1. Focus on cost-effectiveness: Evaluate the cost-effectiveness of advanced imaging and AI technologies, particularly in resource-limited settings, to assess their long-term feasibility.
2. Refine AI algorithms: Continue refining AI algorithms to improve specificity in detecting lobular carcinoma and develop AI tools that integrate genomic, clinical, and imaging data for personalized treatment plans.
3. Address ethical and practical issues: Explore ethical concerns such as AI overreliance, bias, and data privacy and address practical barriers like clinician training and user-friendly AI interfaces.
4. Investigate emerging technologies: Study emerging technologies like AI-enhanced radiomics, hybrid imaging systems, and 3D ultrasound, which could improve diagnostic accuracy and require validation through clinical trials.

## 8 Areas for further research

- AI and radiomics integration: Research the potential of AI combined with radiomics to improve imaging accuracy, treatment response prediction, and prognosis.
- Longitudinal AI studies: Conduct long-term studies to assess how AI impacts patient outcomes, diagnostic accuracy, and clinical decision-making in real-world environments.

## 9 Conclusions

LC is challenging to diagnose due to its subtle imaging characteristics, but advancements in imaging modalities hold promise for improving early detection and outcomes. While mammography and ultrasound remain important for initial assessments, advanced techniques like MRI, DBT, CEM, and BSGI offer higher diagnostic accuracy, particularly in dense breast tissue and high-risk patients. Integrating these methods, along with emerging technologies like AI and radiomics, into clinical workflows has significant potential to improve LC diagnosis. However, this integration requires careful planning and collaboration among clinicians, radiologists, and researchers to optimize diagnostic pathways and enhance patient outcomes.
